# Off-axis points encoding/decoding with orbital angular momentum spectrum

**DOI:** 10.1038/srep43757

**Published:** 2017-03-08

**Authors:** Jiaqi Chu, Daping Chu, Quinn Smithwitck

**Affiliations:** 1 Centre for Photonic Devices and Sensors, University of Cambridge, 9 JJ Thomson Avenue, Cambridge CB3 0FA, UK; 2 Disney Research, Glendale, California 91201-5020, USA

## Abstract

Encoding/decoding off-axis points with discrete orbital angular momentum (OAM) modes is investigated. On-axis Laguerre-Gaussian (LG) beams are expanded into off-axis OAM spectra, with which off-axis points are encoded. The influence of the mode and the displacement of the LG beam on the spread of the OAM spectrum is analysed. The results show that not only the conventional on-axis point, but also off-axis points, can be encoded and decoded with OAM of light. This is confirmed experimentally. The analytical result here provides a solid foundation to use OAM modes to encode two-dimensional high density information for multiplexing and to analyse the effect of mis-alignment in practical OAM applications.

Optical encoding/decoding, transmission of data and rendering of information have benefited from multiplexing/demultiplexing schemes. Taking advantage of light’s various degrees of freedom, there have been time-division^1^,^2^, polarization-division^3^ and wavelength-division^4^,^5^ multiplexing, based on which transmission capacity can be increased.

The last few decades have seen the use of orbital angular momentum (OAM) as an additional degree of freedom in the transfer of information^6^,^7^,^8^,^9^,^10^,^11^. It has been known from the Maxwell’s theory that electromagnetic fields can carry both energy and momentum. An exchange of momentum can involve linear momentum and angular momentum. Poynting^12^ suggested that circularly polarized light should have angular momentum, which was demonstrated by Beth^13^ with the rotation motion of a birefringent wave plate. This spin component of angular momentum arising from spin of photons has a value of ±ħ per photon. Multiple units of ħ are required for higher-order transitions^14^,^15^, which are corresponding to the other component of angular momentum, OAM. In the 1990s^16^, helically phased beams were experimentally generated and shown to possess a well-defined OAM of multiple of ħ.

While polarization-division multiplexing can double the transmission capacity with two polarization states^17^, OAM mode-division multiplexing can potentially increase the capacity greatly with theoretically unbounded state space of OAM. By encoding and decoding data as amplitude of OAM beams, capacity of optical links can reach Gbits/s, and Tbits/s when orthogonality of OAM modes is used in combination with other degrees of freedom^7^,^8^.

So far, OAM beams that conserved rotational symmetry and used for OAM multiplexing were considered to be reliable only in on-axis communication^18^. Inasmuch as information of only the on-axis point is encoded with an OAM beam, and off-axis points are not coded, information density coded with OAM beams is hitherto limited. In some applications, encoding and decoding information should not be subject to zero-dimensional (point) coding^19^,^20^,^21^. Therefore, we investigated the possibility of encoding and decoding information with OAM two dimensionally^22^,^23^. This paper shows how off-axis points are encoded/decoded with an OAM spectrum so that they can be multiplexed in transmission.

## Results

### Encoding an off-axis point with OAM of light

#### Expanding an on-axis Laguerre-Gaussian (LG) beam

In order to analyse misaligned OAM beams, researchers have expanded an off-axis OAM beam into a summation of on-axis OAM beams which suggests a discrete OAM spectrum^24^. From another perspective, we wish to expand an on-axis Laguerre-Gaussian (LG) beam into a summation of OAM beams centred at a different reference axis, which would be helpful to encoding and decoding off-axis points. Here we demonstrate that encoding an on-axis LG beam with an off-axis point is equivalent to encoding a discrete OAM spectrum reference to the axis at the off-axis point.

We use an on-axis LG beam, one widely used form of helically phased beams, to encode off-axis points. A LG beam with a radial mode index and an azimuthal mode index is given by Yao^15^. Considering a LG beam with radial mode index 0, the distribution at the beam waist is given by


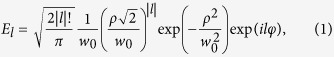


where *l* is the azimuthal mode index, (*ρ, φ*) is the cylindrical coordinate system, and *w*_0_ is the beam waist of the on-axis LG beam.

**Figure 1:**
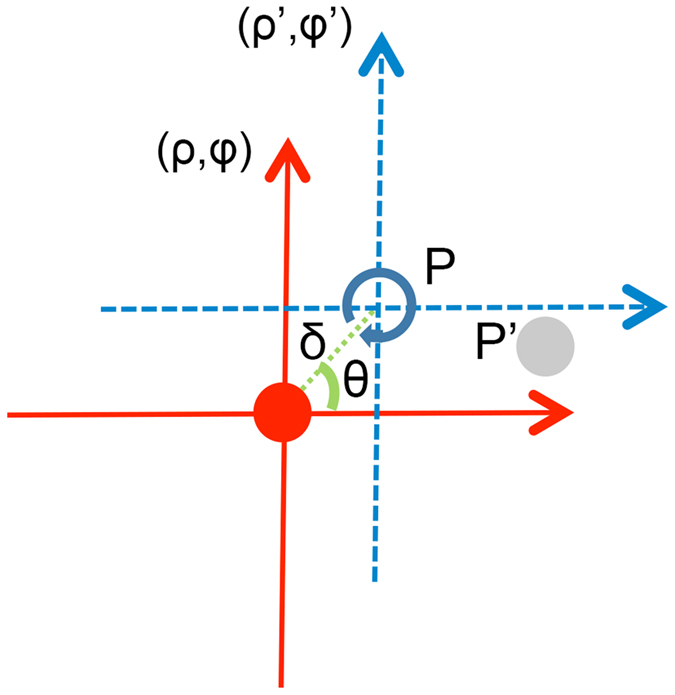
Schematic of coordinate systems. The coordinate system (*ρ, φ*) is the cylindrical coordinate system where the initial encoded on-axis LG beam is described. The displaced coordinate system (*ρ*′, *φ*′) is centred at the off-axis point P, which is described as (*δ, θ*) in the original coordinate system. P′ is another off-axis point to be coded with the on-axis LG beam.

Figure 1 shows the plane transverse to the propagation direction of a LG beam at the beam waist. The point P is an off-axis point to be encoded and decoded, which is also the origin of the (*ρ*′, *φ*′) cylindrical coordinate system. We expand an on-axis LG beam referenced at the original coordinate (*ρ, φ*) into an off-axis OAM spectrum referenced to an off-axis point P. The spiral term can be expanded using binomial decomposition. Because of the Fourier relationship between the angle and OAM^25^, the Gaussian term can be expanded into an OAM spectrum comprising infinite modes. An on-axis LG beam can therefore be written as a weighted summation of infinite off-axis OAM beams


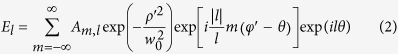


for on-axis LG beams with azimuthal mode indices *l* ≠ 0, where


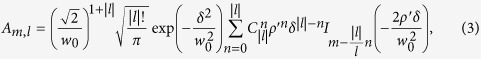


and


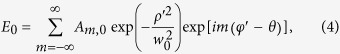


for the on-axis Gaussian beam, where


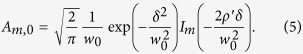


In the equations, 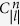 are binomial coefficients, and *I*_*m*_(*x*) is the modified Bessel function of the first kind.

#### Encoding an off-axis point with an OAM spectrum

Equations (2) and (3) contain a spiral term and a Gaussian term referenced to an off-axis point, which is origin of the coordinate system (*ρ*′, *φ*′). The equations suggest that a LG beam as defined on-axis can be considered to possess an infinite set of OAM modes as referenced to a different axis. Reference to the off-axis point P, there is always an OAM spectrum comprising infinite modes *m* representing the on-axis LG beam. The on-axis LG mode *l* can be expanded into a superposition of off-axis LG modes *m* as


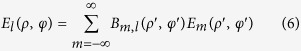


where


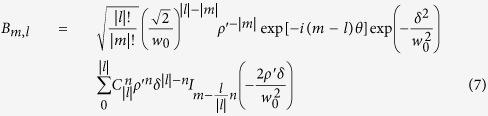


for *l* ≠ 0, and


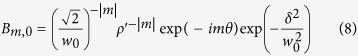


for *l* = 0.

Therefore, encoding an off-axis point with an on-axis LG beam can be achieved by encoding the off-axis point with an OAM spectrum. The amplitude of an expanded OAM beam in the spectrum is rotationally symmetric. It is a function of charge *l* and beam waist *w*_0_ of the on-axis LG beam, and displacement *δ* of the off-axis point.

**Figure 2:**
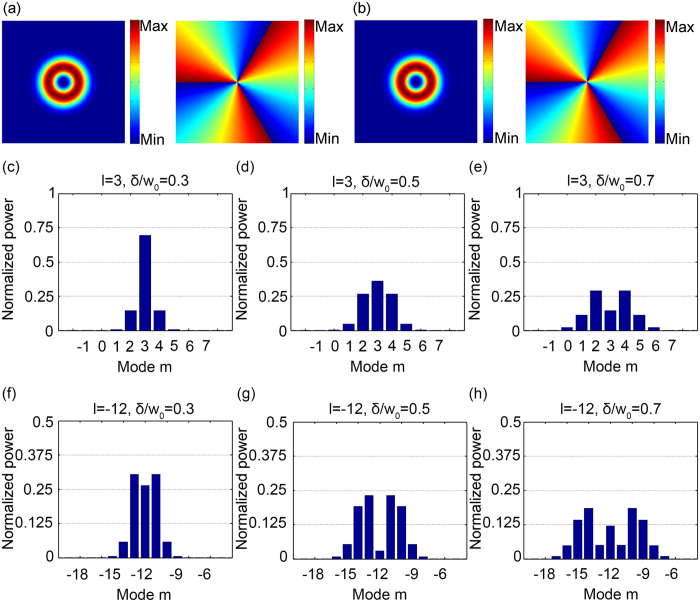
(**a**) Amplitude and phase profiles of an on-axis LG beam with the azimuthal mode index *l* = 3. (**b**) Amplitude and phase profiles of the reconstructed beam summed from expanded OAM beams corresponding to modes *m* = −1 to 7. (**c–e**) Normalized power of expanded modes *m* = −1 to 7 for the on-axis LG beam with azimuthal mode index *l* = 3. (**f–h**) Normalized power of expanded modes *m* = −18 to −6 for the on-axis LG beam with azimuthal mode index *l* = −12.

Figure 2(a) shows amplitude and phase profiles of an on-axis LG beam with mode *l* = 3. Figure 2(b) shows amplitude and phase profiles of the summation of nine largest components ranging from *m* = −1 to 7 of the expanded OAM beams, which confirms the expansion is a good representation.

Spread of an OAM spectrum changes with (1) the original on-axis LG mode *l*, and (2) ratio of displacement of the off-axis point to beam waist of the original on-axis LG mode *δ*/*w*_0_. For different ratio of displacement to beam waist *δ*/*w*_0_, Fig. 2(c–h) shows normalized power of the expanded OAM spectrum for *l* = 3 and *l* = −12 as defined by


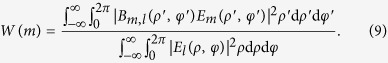


### Reconstructing the off-axis point

Among the encoded OAM spectrum, each spiral phase component can be further expanded back to the (*ρ, φ*) coordinate system. For an on-axis LG beam with azimuthal mode index *l* ≠ 0,


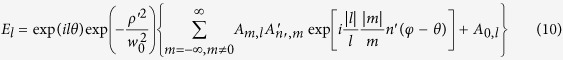


where


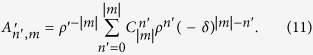


For the on-axis LG beam (Gaussian beam) with azimuthal mode index *l* = 0,


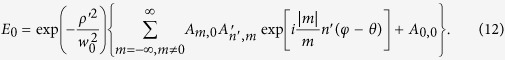


By further expanding the spiral phase terms into an OAM spectrum as defined along the original (*ρ, φ*) coordinate system, eqs (10) and (11) indicate that each phase component in the OAM spectrum that encoded with the off-axis point can be decoded with a summation of on-axis phase components. Decoding the modes *n*′ from 0 to |*m*| for a specified *A*_*m,l*_ would decode the component corresponding to the mode *m*, which contains part of the encoded information. Decoding *A*_*m,l*_ from *m* = −∞ to ∞ would further decode the encoded point entirely. The process is similar to convolution.

As *A*_*m*,*l*_ = *A*_*m*,−*l*_ and 
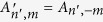
, when a point is encoded with an on-axis LG mode with the mode index +*l* or −*l*, amplitude of each component in the encoded spectrum is the same, while the phase is complementary with respect to each other. By decoding with a complementary on-axis spiral phase characterized by −*l*, each expanded component specified by mode (|*lm*|/*lm*)*n*′, and a complementary component specified by the mode −(|*lm*|/*lm*)*n*′ can be applied to decode one of the components in the encoded spectrum. Summing together, the decoded components would reconstruct the encoded off-axis point. As a result, the off-axis point P can be reconstructed when a spiral phase mode complementary with respect to the encoding mode is applied to decode. The decoding spiral phase should reference to the same axis as the encoding LG beam, with the same number of intertwined helices but different handedness.

### Minimization of noise

#### Decoded spectrum

**Figure 3:**
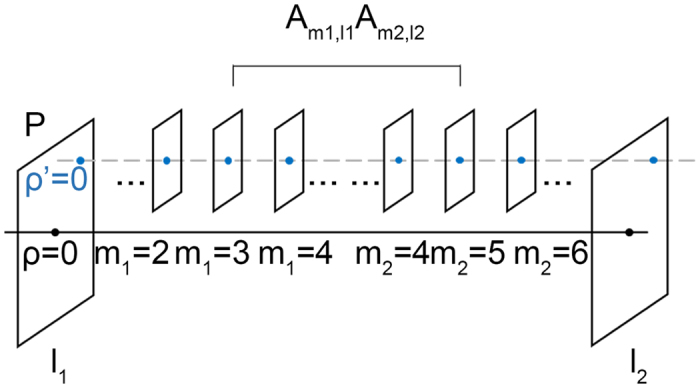
Sketch of an off-axis point P at *ρ*′ = 0 encoded with the on-axis LG beam with spiral phase characterized by azimuthal mode index *l*_1_ and decoding with the on-axis spiral mode *l*_2_. Both of the encoding and decoding LG modes expand a spectrum centred at the off-axis point P, comprising an infinite set of weighted modes *m*_1_, *m*_2_ = −∞ ~ ∞ of the encoding/decoding OAM spectra.

Figure 3 shows the case that an off-axis point P is encoded with the on-axis LG beam with azimuthal mode *l*_1_ and decoding with the on-axis spiral phase with mode index *l*_2_. Both of the encoded and the decoding spiral phase expand a spectrum at the decoding point P, where *ρ*′ = 0. Expanded modes 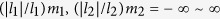 comprise the encoded and decoding OAM spectra.

Each expanded mode 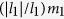 in the encoding OAM spectrum would act with each component 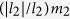 in the decoding OAM spectrum, forming an updated OAM beam with updated OAM mode index 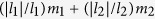. The entire decoded pattern referenced in the (*ρ*′, *φ*′) coordinate system is therefore the summation of the infinite set of updated OAM beams expanded from an on-axis LG mode with updated azimuthal mode index *l* = *l*_1_ + *l*_2_.

#### Noise of encoding/decoding an off-axis point

When an on-axis point is encoded with a LG beam and decoded with a complementary mode, the point is reconstructed. When a non-complementary mode is applied, the amplitude of the on-axis point is 0 because of the orthogonality of OAM modes. However, this is not necessarily the case when an off-axis point is encoded.

**Figure 4:**
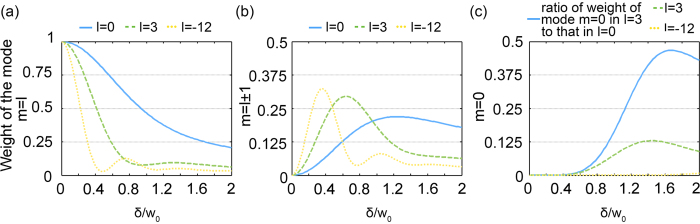
Normalized power of the expanded off-axis OAM modes (**a**) *m* = *l*, (**b**) *m* = *l* ± 1, and (**c**) *m* = 0 for the on-axis LG beam of the OAM modes *l* = 0, 3 and −12, respectively.

In an expanded OAM spectrum, each mode *m* corresponds to an OAM beam. Figure 4 shows the change of weight of selected expanded modes with respect to different displacement and on-axis LG beams. The amplitude of the OAM beam is a vortex or a Gaussian distribution, with the inner radius increasing with the absolute value of the mode index |*m*|. At point P, the OAM beam with mode index *m* = 0 would introduce noise. Figure 4(c) shows the normalized power of the OAM beam corresponding to the mode *m* = 0 in the OAM spectrum of the on-axis LG beams *l* = 0, 3, and −12. For a fixed updated azimuthal mode index 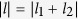 and beam waist of an on-axis LG beam, the noise introduced by the expanded mode *m* = 0 increases when the ratio of the displacement increases.

Additionally, as the absolute azimuthal mode index of the on-axis LG beam 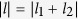 increases, the weight of the expanded OAM mode *m* = 0 decreases. Therefore, when decoded by a non-complementary on-axis mode, a larger absolute value of the encoding and decoding mode 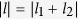 would also minimize the noise at the coded point P.

### Encoding/decoding multiple points

When multiple points are encoded and decoded, the entire OAM spectrum would contribute to noise. Though an OAM spectrum comprises infinite modes theoretically, some of them have smaller weight and are less effective. Because each expanded mode is corresponding to a vortex, the condition of a different off-axis point can be encoded and decoded is that the point is placed within all of the effective vortices. As inner radius of the vortices decreases with decreased absolute value of the expanded mode |*m*|, the largest displacement of off-axis points that can be encoded and decoded depends on the smallest effective mode |*m*_*e*_|.

**Figure 5:**
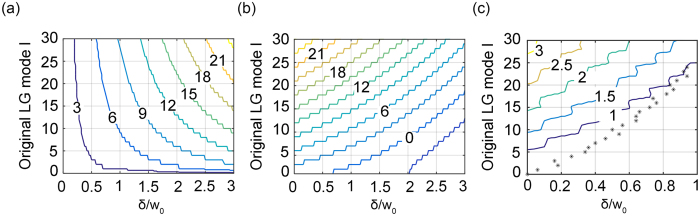
(**a**) Width of spectra that covers 90% of the entire energy. (**b**) The smallest effective expanded mode. (**c**) The contour shows largest displacement of an-off-axis point that can hide in the effective expanded vortices and the star symbols show the largest displacement which an on-axis LG mode can encode/decode with ≤10% of the crosstalk noise from another off-axis points.

Here we consider the expanded modes effective if they are near the mode *m*=*l* and summation of their energy covers 90% of the entire energy. For different original on-axis LG modes *l* and ratio of displacement to beam waist *δ*/*w*_0_, Fig. 5(a) shows width of an OAM spectrum |*l* − *m*_*e*_|+ 1. Based on the width, Fig. 5(b) shows the smallest effective mode *m*_*e*_. When *m*_*e*_ > 0, it creates a crosstalk vortex where off-axis points can hide in so that they can be encoded and decoded. In Fig. 5(c), the contour shows the largest displacement of an off-axis point that can hide in the crosstalk vortex in terms of its ratio to the beam waist of the original on-axis LG beam. The largest displacement is defined at the original (*ρ, φ*) coordinate system, and the inner radius of an expanded vortex is defined at the first 1/*e* of the maximum amplitude. Using this ratio one can find the largest displacements of off-axis points that can be encoded and decoded with ≤10% of the crosstalk noise introduced from another off-axis point encoded by different on-axis LG mode *l*, and they are represented by the star symbols in Fig. 5(c).

### Encoding/decoding images

**Figure 6:**
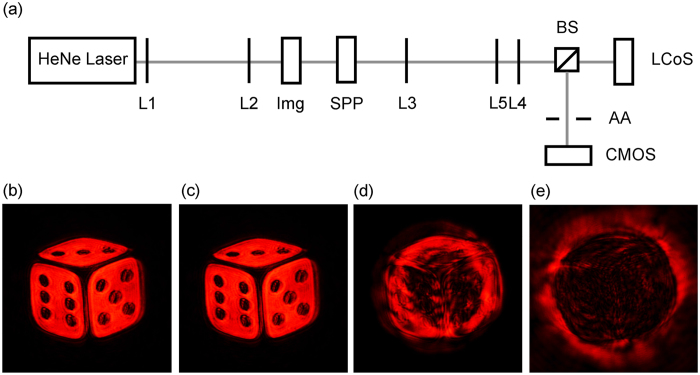
(**a**) Optical Setup. (**b**) Projected image without encoding/decoding. (**c–e**) Recorded intensity when the image is encoded with on-axis spiral phase plate *l*_1_ = −8 and decoded with *l*_2_ = +8, +16, and +29, respectively.

A two-dimensional (2D) image contains an on-axis point and multiple off-axis points. Coding multiple off-axis points with OAM spectra allows encoding and decoding of 2D images. Figure 6(a) shows the experimental setup that is used to encode and decode a 2D image. Two lenses (L1 and L2) are used to expand light emitting from a HeNe laser. An image mask (Img) imprints a 2D image onto the projected beam. This image is then encoded with an OAM mode by a fixed spiral phase plate^26^,^27^ (SPP) and decoded by another OAM mode generated on a phase-only liquid crystal on silicon (LCoS) spatial light modulator (SLM). Lenses L3 and L4 are used to relay the beam and the lens L5 is used to project the 2D image. In order to ensure perpendicular incidence to the LCoS SLM, a beam splitter (BS) is used. Because the light diffracted from the LCoS SLM contains high orders, an adjustable aperture (AA) is used to filter out them and keep the first diffraction order only. The intensity profiles of the decoded patterns are recorded using a CMOS image sensor chip directly.

Figure 6(b) is the projected image with encoding or decoding. Figure 6(c) is the reconstructed image encoded with an on-axis OAM mode *l*_1_ = −8 and decoded with the complementary on-axis OAM mode *l*_2_ = +8. When the image-carrying OAM beam is decoded with a non-complementary OAM mode, the decoded image contains bright patterns in the vortices, as shown in Fig. 6(d–e). As the reconstructed image would appear at the vortices, these bright patterns are crosstalk introduced by smaller expanded modes in the coded OAM spectrum. The crosstalk can be reduced by increasing the mode separation, i.e. the summation *l* = |*l*_1_ + *l*_2_|. In Fig. 6(d), the summation of mode indices *l* = |*l*_1_ + *l*_2_| is not large enough to spatially remove crosstalk terms from a reconstructed image. While in Fig. 6(e), the updated OAM mode is large enough to separate the reconstructed image and the vortex, which indicates that encoding 2D information using OAM modes is promising for multiplexing in a single optical channel. Note that although the OAM beam we generated in this experiment is not a pure LG beam, the observed trend confirms to that shown in Fig. 5(c).

## Discussion

In this study, we confirmed that not only the on-axis point, but also off-axis points can be encoded and decoded with OAM under certain conditions. By expanding an on-axis LG beam into an off-axis OAM spectrum, an off-axis point is encoded with the OAM spectrum. Each OAM beam in the expanded OAM spectrum is then further expanded back into an on-axis OAM spectrum, which suggests that the off-axis point can be reconstructed using an on-axis OAM beam when it is encoded with an on-axis LG beam. Modes with the same number of helices but opposite handedness are complementary modes to encode and reconstruct a point.

We further investigate the OAM spectrum when an off-axis point is encoded for multiplexing purpose. OAM multiplexing benefits from orthogonality of theoretically unlimited number of different OAM modes and has potentially unlimited information capacity. In the traditional case, where an on-axis point is encoded and decoded, the orthogonality of OAM modes makes the amplitude at the encoded point become zero when the point is decoded with a non-complementary OAM mode. In the case of encoding/decoding an off-axis point, the amplitude cannot be rigorously zero. Because the expanded OAM spectrum comprises an infinite modes, there are always infinite pairs of expanded modes *m*_1_ and *m*_2_ in the encoding/decoding spectra which fulfil (|*l*_1_|/*l*_1_)*m*_1_ + (|*l*_2_|/*l*_2_)*m*_1_ = 0 and introduce crosstalk at the encoded point. When decoding OAM spectrum is chosen to be separable from the encoding OAM spectrum, the weight of the crosstalk components will be sufficiently small, and a reconstructed off-axis point can be separable from the crosstalk due to multiplexing of the signals generated by off-axis points encoded by different OAM modes.

We evaluate the crosstalk via relative power terms. Spread of an OAM spectrum changes with the expanded LG mode, as well as the off-axis displacement from the origin. We find that larger separation of encoded/decoded modes, and smaller ratios of off-axis displacement to the beam waist would reduce the crosstalk.

When multiple points are encoded and decoded, the entire OAM spectrum which comprises infinite terms would contribute to crosstalk. The crosstalk terms are off-axis vortices. Because the energy of an expanded OAM spectrum is centralized, hiding off-axis points within inner radius of effective crosstalk vortices would allow OAM spectra for multiplexing. The displacement of off-axis points that can be encoded/decoded increases as the sum of the encoding/decoding LG modes increases. For absolute summations up until |*l*_1_ + *l*_2_| = 30, we analysed the maximum displacements that can be encoded and decoded in terms of their ratio to the beam waist.

Experimentally we encode a 2D image with an on-axis spiral phase plate of a given mode, and decode the encoded image with on-axis spiral phase plates of different modes. The image is reconstructed when it is decoded by a complementary mode. When decoded by a non-complementary mode, it shows a noisy image due to crosstalk and the level of crosstalk reduces as the sum of the encoding and decoding mode indices increases, which confirms the theory.

All together the mathematical derivations and experimental findings reveal that off-axis image points can be encoded and reconstructed with appropriate OAM modes. Choosing encoding/decoding LG modes and displacement of off-axis points carefully allow encoding of different OAM modes for multiplexing in a single transmission channel with minimum crosstalk in the reconstructed images.

Furthermore the development in rendering and transmitting information can contribute to the areas such as communications and image displays. In communications, OAM modes stand out as an additional degree of freedom in increasing channel capacity with theoretically infinite number of different modes. The data encoded with OAM modes can now be a temporal point or a 2D array. For displays, three-dimensional (3D) images can be mimicked by showing multiple 2D images simultaneously for 3D perceptions. Without reducing the resolution of the images, 3D displays have benefited from polarization multiplexing by using the two polarization states to encode two views of 2D images. With more views of 2D images encoded with different OAM modes, it is expected that smoother parallax and high resolution can be achieved at the same time.

Finally our results can also help to understand the mis-alignment effect in different practical applications, such as the coupling of OAM modes to an off-axis plasmonic structure^28^ and to bounded electrons^29^.

## Methods

### Expanding an on-axis LG mode into an OAM spectrum

The coordinate system contains N × N = 1001 × 1001 points (Fig. 2a,b). Energy of the original LG beam (Fig. 2a) is normalized according to the normalization constant as described in eq. (1).

### OAM spectrum analysis

25 expanded modes *m* for Fig. 4 and 21 for Fig. 2c–h are calculated to show that the sum of energy of the components in the spectrum equals that of the original LG beam.

### Original 2D images

The original 2D image is a chrome mask imprinted on glass. The imprinted dice is divided in 400 × 400 pixels, with a pixel size 5 micron.

### Laser

We use a 632.8 nm linearly polarized HeNe laser. The power is 5 mW and the beam diameter is 0.81 mm.

### Generation of OAM beam

We generate an OAM beam by the use of a commercially available spiral phase plate. A spiral phase plate is a transparent phase modulation device with its thickness increases with azimuthal angle^26^,^27^.

### Reconstructed/decoded 2D images

A liquid crystal on silicon (LCoS) device assembled in house is used to decode images. The LCoS has 1280 × 720 pixels with a pixel pitch of 15 μm.

### Recoding intensity profiles

Intensity profiles of the images and decoded patterns are directly recorded using a CMOS sensor from Nikon D7000. All the intensity profiles are recorded using the same imaging setup.

## Additional Information

**How to cite this article:** Chu, J. *et al*. Off-axis points encoding/decoding with orbital angular momentum spectrum. *Sci. Rep.* **7**, 43757; doi: 10.1038/srep43757 (2017).

**Publisher's note:** Springer Nature remains neutral with regard to jurisdictional claims in published maps and institutional affiliations.
